# Gesture Recognition Using Surface Electromyography and Deep Learning for Prostheses Hand: State-of-the-Art, Challenges, and Future

**DOI:** 10.3389/fnins.2021.621885

**Published:** 2021-04-26

**Authors:** Wei Li, Ping Shi, Hongliu Yu

**Affiliations:** Institute of Rehabilitation Engineering and Technology, University of Shanghai for Science and Technology, Shanghai, China

**Keywords:** hand gesture recognition, prosthesis hand, deep learning, pattern recognition, convolutional neural network, recurrent neural network, surface electromyography

## Abstract

Amputation of the upper limb brings heavy burden to amputees, reduces their quality of life, and limits their performance in activities of daily life. The realization of natural control for prosthetic hands is crucial to improving the quality of life of amputees. Surface electromyography (sEMG) signal is one of the most widely used biological signals for the prediction of upper limb motor intention, which is an essential element of the control systems of prosthetic hands. The conversion of sEMG signals into effective control signals often requires a lot of computational power and complex process. Existing commercial prosthetic hands can only provide natural control for very few active degrees of freedom. Deep learning (DL) has performed surprisingly well in the development of intelligent systems in recent years. The significant improvement of hardware equipment and the continuous emergence of large data sets of sEMG have also boosted the DL research in sEMG signal processing. DL can effectively improve the accuracy of sEMG pattern recognition and reduce the influence of interference factors. This paper analyzes the applicability and efficiency of DL in sEMG-based gesture recognition and reviews the key techniques of DL-based sEMG pattern recognition for the prosthetic hand, including signal acquisition, signal preprocessing, feature extraction, classification of patterns, post-processing, and performance evaluation. Finally, the current challenges and future prospects in clinical application of these techniques are outlined and discussed.

## 1. Introduction

For human, the worth of hand is indisputable. The hand is the most diverse and dexterous part of the human body, which can execute various activities to interact with the environment by adopting a variety of different motion strategies (Feix et al., 2016; Sartori et al., 2016). With the rapid development of physiology, anatomy, mechatronics, and software, various types of prosthetic hands have appeared in the market (Jiang et al., 2014a; Amsuess et al., 2016; Nissler et al., 2016), such as i-Limb hand (van der Niet et al., 2013), SmartHand (Cipriani et al., 2011a), Michelangelo hand (Luchetti et al., 2015), iCub hand (Schmitz et al., 2010), and HIT/DLR prosthetic hand (Butterfass et al., 2001). These prosthetic hands have been developed with more and more degrees of freedom (DOFs) to achieve the goal of completing the amputees' activities of daily living. However, because of the restriction of human–computer interaction (HCI), the existing prosthetic hands has great difficulty in reproducing the flexibility and function of biological hands and the life of hand amputees is still very difficult (Farina and Aszmann, 2014; Ortiz-Catalan et al., 2015; Chadwell et al., 2016). Recently, biological signals, which contain abundant information about the human body's motion intention, have shown broad prospects in HCI field. Specifically, surface electromyography (sEMG) signals reflects information of upper limb neuromuscular system, which has great potential for controlling prosthetic hand (Saponas et al., 2008; Khushaba et al., 2012; Farina et al., 2014a).

**Figure 1:**
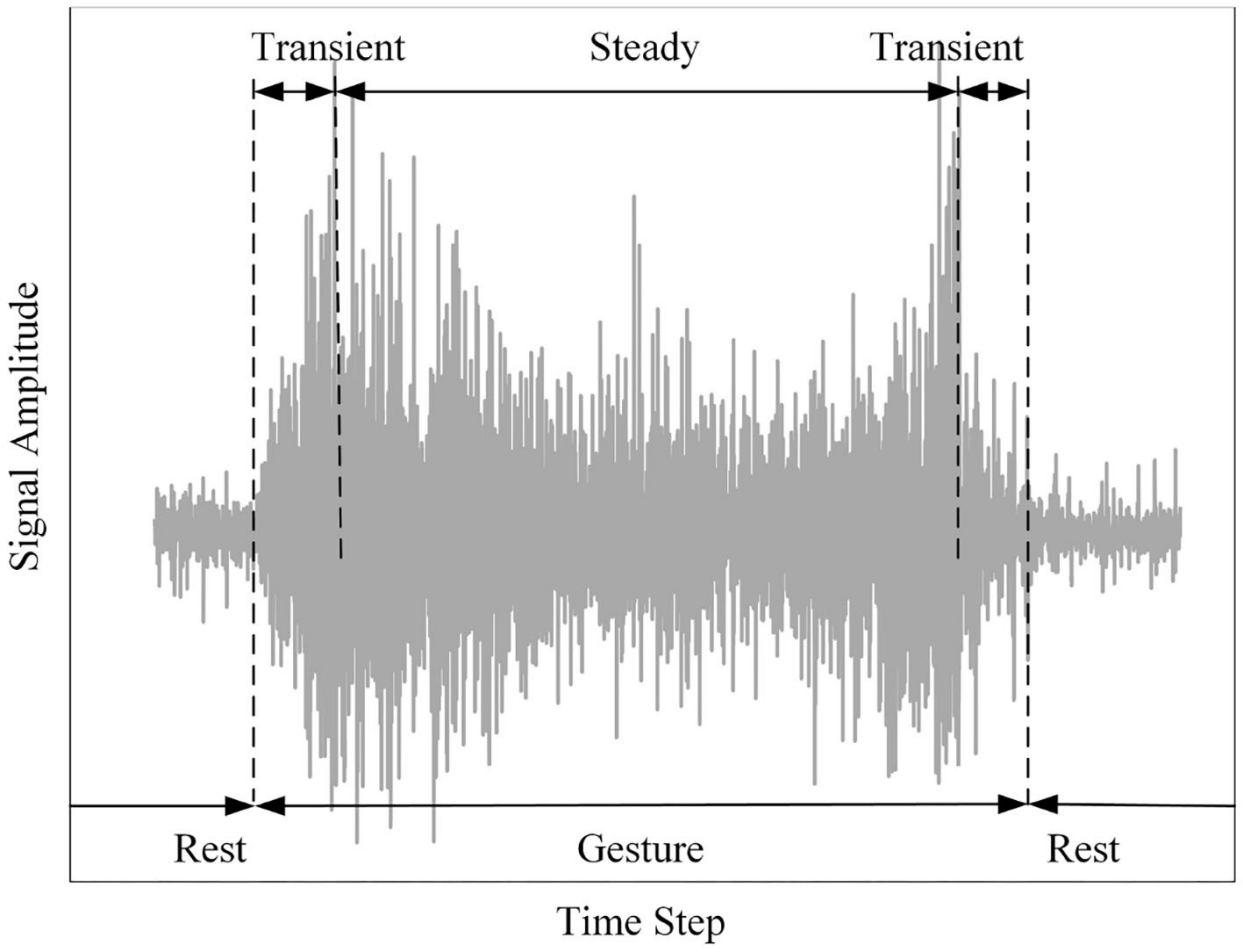
The surface electromyography (sEMG) data of a gesture.

When skeletal muscles are activated by physiological neural activity, sEMG records internal muscle's electrical activity from the surface of the skin and thus reflects the generation and propagation of composite action potentials, which is the collective action of motor units (Scheme et al., 2011; Farina and Aszmann, 2014; Geng et al., 2016; Athavale and Krishnan, 2017). sEMG control of prosthetic hands refers to a set of techniques of extracting the available information from sEMG signal of the upper limb and applying it to drive external device. The process of hand movement is not instantaneous. With regard to hand movements, skeletal muscle contraction can be mainly defined as two types: dynamic and static. The former involves the change of muscle fiber shape and the motions of upper limb joint, while the latter involves the invariance of muscle fiber and upper limb joint (Englehart et al., 2001; McGill, 2004; Gusman et al., 2017) (see Figure 1). Corresponding, the sEMG signals can be regarded as a random process and can be termed as two states: transient and steady state, which can be used for detecting the motion classes (Hudgins et al., 1993; Atzori et al., 2014).

The prosthetic hand is a product of human centered design. Since sEMG signal is bioelectrical signal generated by the interaction between human body and nervous system, the mode of sEMG signal depends on both the user's subjective intention and the interaction environment. Understanding how humans use their hands and how prosthetic hands are used in daily life is the key to designing prosthetic hands. The upper limbs of the human body, including the upper arm, elbow, forearm, wrist, and hand, have 20+ DOFs. The muscle mass of the upper limb is usually small and slender, and the muscle strength is relatively weak, so the sEMG signal amplitude of the upper limb is small, which increases the difficulty of sEMG analysis. It is still unrealistic to recognize flexible upper limb motion based on sEMG signal of several muscles, so it is necessary to simplify and reconfigure the upper limb movement during activities of daily living (Yang et al., 2014). At present, the focus of recognition is the hand movement. Among the taxonomies proposed so far, the GRASP taxonomy is a widely recognized classification method, which is directly for the study of hand movement (Feix et al., 2009, 2016). The hand grasping is divided into 33 categories according to four parameters, including thumb position, power type, opposition type, and virtual finger assignments. On the basis of activities of daily living, work operation needs, kinematic state, and force distribution (Bullock et al., 2013; Vergara et al., 2014; Abbasi et al., 2016; Llop-Harillo et al., 2019) proposed several similar dominant gesture categories, such as cylindrical grasp, oblique palmar grasp, hook grasp, lumbrical grasp, power-precision grasp, precision grasp, pinch grasp, tripod, pulp pinch, lateral pinch, and non-prehensile grasp. According to the way of interaction with external objects, Averta et al. (2020) divided upper limb movements into three categories: intransitive, i.e., gestures, transitive, i.e., motion interacting with an object, tool-mediated, i.e., motion of interaction between objects.

Generally, prosthetic hand has made significant progress, which can meet the basic needs of amputees. However, most control strategies follow the same operating principle: non-pattern recognition and pattern recognition. The traditional non-pattern recognition methods are usually used and limited to on/off control, threshold control, and proportional control (Belter et al., 2013; Hong Liu et al., 2016). sEMG pattern recognition techniques have broken through the limitation of non-pattern recognition and increased the dexterity of prosthetic hand. sEMG pattern recognition is to extract multi-dimension features from sEMG signals, rather than relying entirely on EMG amplitude, which is usually used in non-pattern recognition. A mature prosthetic hand design includes motion patterns and related motion trajectories. Thus, the control algorithm needs parameters, such as motion mode, motion orientation, and kinematics. The pattern in sEMG contains abundant information about the motion intention. Once the sEMG signals of the expected motion intention are classified by pattern recognition, the prosthetic hand would receive the command to perform the corresponding action. Therefore, pattern recognition techniques can make it easier for amputees to control their prosthetic hands.

To transform the complex and highly variable information of sEMG signal into useful control signal of prosthetic hands, advanced data analysis and pattern recognition techniques that can describe and analyze big data are needed. The sEMG pattern recognition techniques can be achieved with two primary processing methods: machine learning (ML) and deep learning (DL). ML, which is the method based on feature engineering, could learn and perform tasks from the data input of automatic modeling. For conventional machine learning algorithms, there are limitations in processing raw data, because they could not effectively train on inconsistent, noisy, abstract, and high-dimensional data (Wang et al., 2019). Meanwhile, a large number of studies show that the performance of sEMG pattern recognition relies heavily on heuristic hand-crafted feature. DL, which is the method based on feature learning, is a branch of machine learning. The feature of DL lies in its hierarchical model architecture. The model architecture with deep layers could extract high-level feature information in multiple representative layers and hidden layers (Côté-Allard et al., 2020). These computational models allow feature extraction and model building procedures to proceed simultaneously, so that features can be learned automatically without hand-crafted, which is more suitable for complex gesture recognition (LeCun et al., 2015; Naik et al., 2016). It has performed excellently well in the development of intelligent systems, including image recognition, machine translation, speech recognition, and automatic driving. In recent years, more and more physiological signal analysis and processing have begun to take advantage of DL, which motivated the study of DL in pattern recognition of sEMG (Atzori et al., 2016; Du et al., 2017; Zhai et al., 2017). Parajuli et al. (2019) reviewed the application of machine learning in sEMG pattern recognition of prosthetic hand. It found that almost all the data used by traditional ML methods come from steady-state signals, but the sEMG signals generated by gestures in daily life are transient. Transient-state analysis is a very challenging task suitable for DL processing.

Although pattern recognition techniques have been applied to sEMG research for decades, DL has only been applied in recent years. With the emergence of large sEMG data sets and the latest development of optimization algorithm, DL has shown brilliant prospects in the field of sEMG pattern recognition for prosthetic hand. At present, there are many basic researches on gesture recognition based on sEMG and DL. However, as far as we know, there is no literature review on these gesture recognition models. Based on the investigation of related papers in recent years, this review makes a comprehensive analysis of the application of DL techniques in the field of prosthetic hand based on sEMG, including the commonly used DL model for upper limb motion intention prediction, the advantages of DL model, and the performance level for system verification. The contribution of this review is mainly in three aspects: (1) we comprehensively analyze the overall structure of DL in sEMG-based gesture recognition; (2) we comprehensively review the latest methods and technologies of sEMG based gesture recognition; (3) we raise the existing challenges and promising research prospects of sEMG based on motion intention recognition in prosthetic hand field.

## 2. Pattern Recognition-Based sEMG

**Figure 2:**
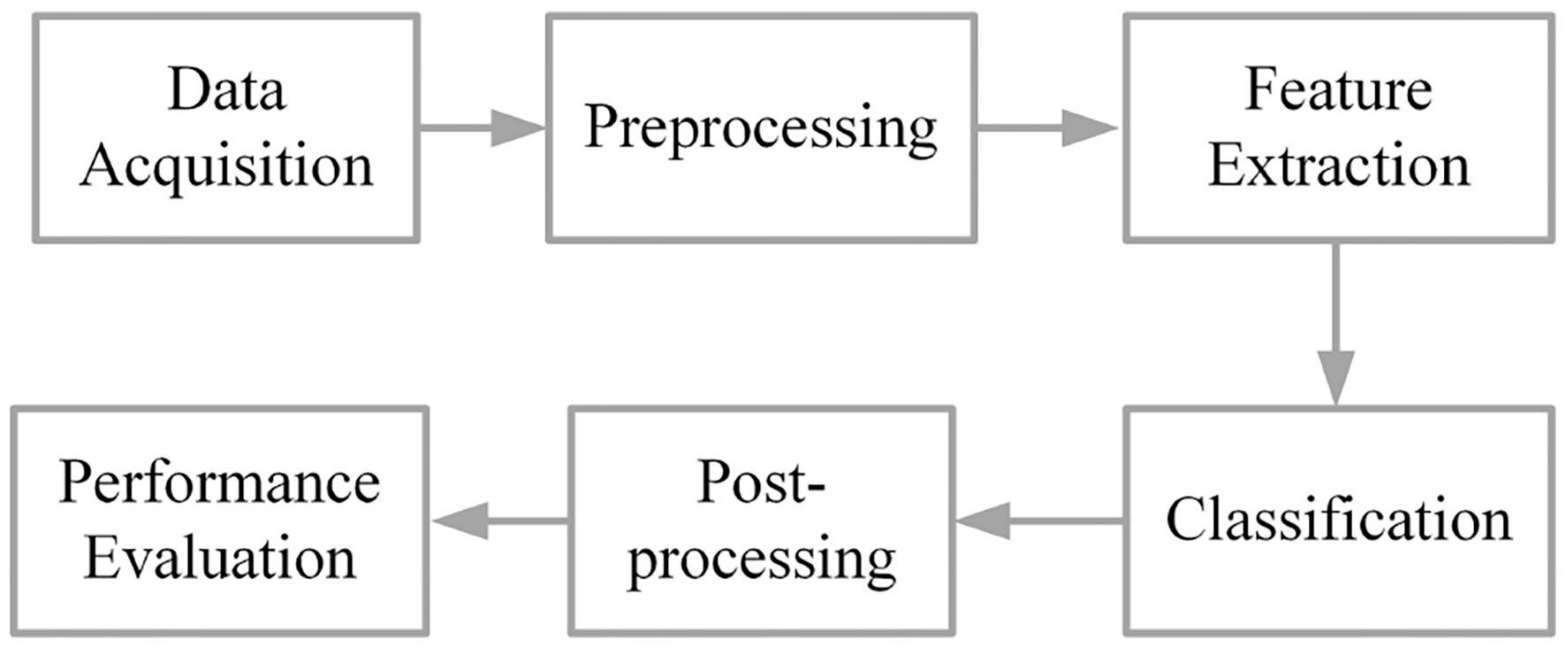
The six stages of the standard structure of the model.

The approaches of gesture recognition model are very similar, but the method of each stage is different; in addition, not all studies use all stages of standard structure. Considering this, we propose a standard approach composed of six parts, which are data acquisition, data preprocessing, feature extraction, classification of patterns, post-processing, and performance evaluation. Figure 2 shows the six stages of the standard approach of sEMG signal pattern recognition based on DL.

### 2.1. Data Acquisition

The sEMG signals are collected from sEMG sensors, which could adopt two different acquisition criteria, sparse multi-channel sEMG and high-density sEMG (HD-sEMG), in the density of the employed electrodes of view (Costanza et al., 2007; Saponas et al., 2010; Patricia et al., 2014).

The sparse multi-channel sEMG generally takes an accurate anatomic localization strategy over the muscle (Castellini et al., 2009). In this configuration, only several paired sensors are placed over the muscles, so gesture labels are usually assigned to sEMG. NinaPro is a typical public database for this configuration, which contains most gestures needed in daily life (Atzori et al., 2014). And there are two modeling methods: extracting feature combination in the light of time window or temporal modeling based on sequence information. HD-sEMG is a dense sampling approach, which is the recording of a high channel count of spatially and densely distributed sEMG electrodes and allows measurement of the spatial distribution of motor unit action potentials. Using this technique on, and around, the forearm allows the detection and classification of multiple muscles interaction as present in gesture recognition problems with a single easy-to-install array of electrodes (Fukuda et al., 2003; Tenore et al., 2009; Li et al., 2010b). CapgMyo and csl-hdemg are two typical open databases of HD-sEMG (Amma et al., 2015; Geng et al., 2016). In this category, the instantaneous value can represent the measured value of a specific muscle activity during the transformation process, so the sEMG signal window can be converted into feature vector or HD-sEMG map. The spatiotemporal distribution of sEMG activity can be gotten.

Both categories have advantages and disadvantages. Sparse multi-channel sEMG have less data to transfer and low cost of hardware resources; however, it is very sensitive to the domain change of sEMG signal, which is the inherent characteristic of sEMG signal. In contrast, HD-sEMG captures the spatial and temporal distribution of motor unit action potentials in muscles through a two-dimensional array of electrodes. This technique increases the amount of data collected and has high recognition efficiency and control quality. For DL, the final prediction results are immediately affected by the quality and quantity of inputs, so the application of HD-sEMG seems to be an effective method to solve myoelectric control problems (Donovan et al., 2018; Nougarou et al., 2018; Tam et al., 2020). However, the increase in the number of electrodes means more complex analog front-end and processing facilities, and additional computing requirements brought by processing algorithms, which depend on the development of hardware devices.

### 2.2. Pre-processing

The sEMG signal is a multi-channel biomedical signal, its data recording is easily affected by the external environment, biological tissue interference, muscle fatigue, electrode displacement, so there is a lot of noise and non-stationarity, which may obscure important information about muscle electrical activity. When the sEMG signals need to be processed by pattern recognition technique, sEMG signals needs to be converted into input signals suitable for feature extraction or DL. Reliable preprocessing techniques are essential for extracting serviceable information in the next step of the analysis. Before motion recognition, the following three steps of preprocessing are usually used: smoothing, normalization, segmentation (Farina et al., 2004, 2014b; Ma et al., 2020).

#### 2.2.1. Smoothing

sEMG is a noisy signal. This means that the probability distribution of the sEMG changes with time. And we can reduce the non-stationarity of sEMG by smoothing, which generally uses the following two steps: rectification and filtering.

Rectification: In this, there is a positive and negative voltage in the sEMG signal, due to the repolarization and depolarization of muscle fibers.

Filtering: It is used to discard noise and extract essential information. Based on the Nyquist–Shannon sampling theorem, since about 95% of the sEMG signal power is concentrated at 400–500 Hz (Clancy et al., 2002; Li et al., 2010a), the lowest sampling frequency of the sensor must be more than twice the highest frequency of the sEMG (Ajiboye and Weir, 2005; Chu et al., 2006). Meanwhile, the filter also uses low-pass filtering method, or moving average method is used, which could be regarded as a special low-pass filter. For moving average method, some features (such as MAV, ARV, or RMS) are computed by windowing the signals, and then to average the features of all channels, or compute directly the features of the average of all channels (Benalcázar et al., 2017).

#### 2.2.2. Normalization

The sEMG signals from different subjects, even different sessions, have enormous differences. In order to compensate for these differences, it is necessary to normalize the data (Ma et al., 2020). Normalization refers to the current amplitude was converted to a percentage of the original or smoothed sEMG amplitude (Clingman and Pidcoe, 2014). The advantage of normalization is that it is simple and accurate, the result has nothing to do with repeatability, and it can improve the accuracy of the model. The disadvantage is that all components analyzed must be fully peaked and separated in the same session (Farina et al., 2010).

#### 2.2.3. Segmentation

The segmentation stage consists of two sections: gesture detection and sliding windowing.

**Figure 3:**
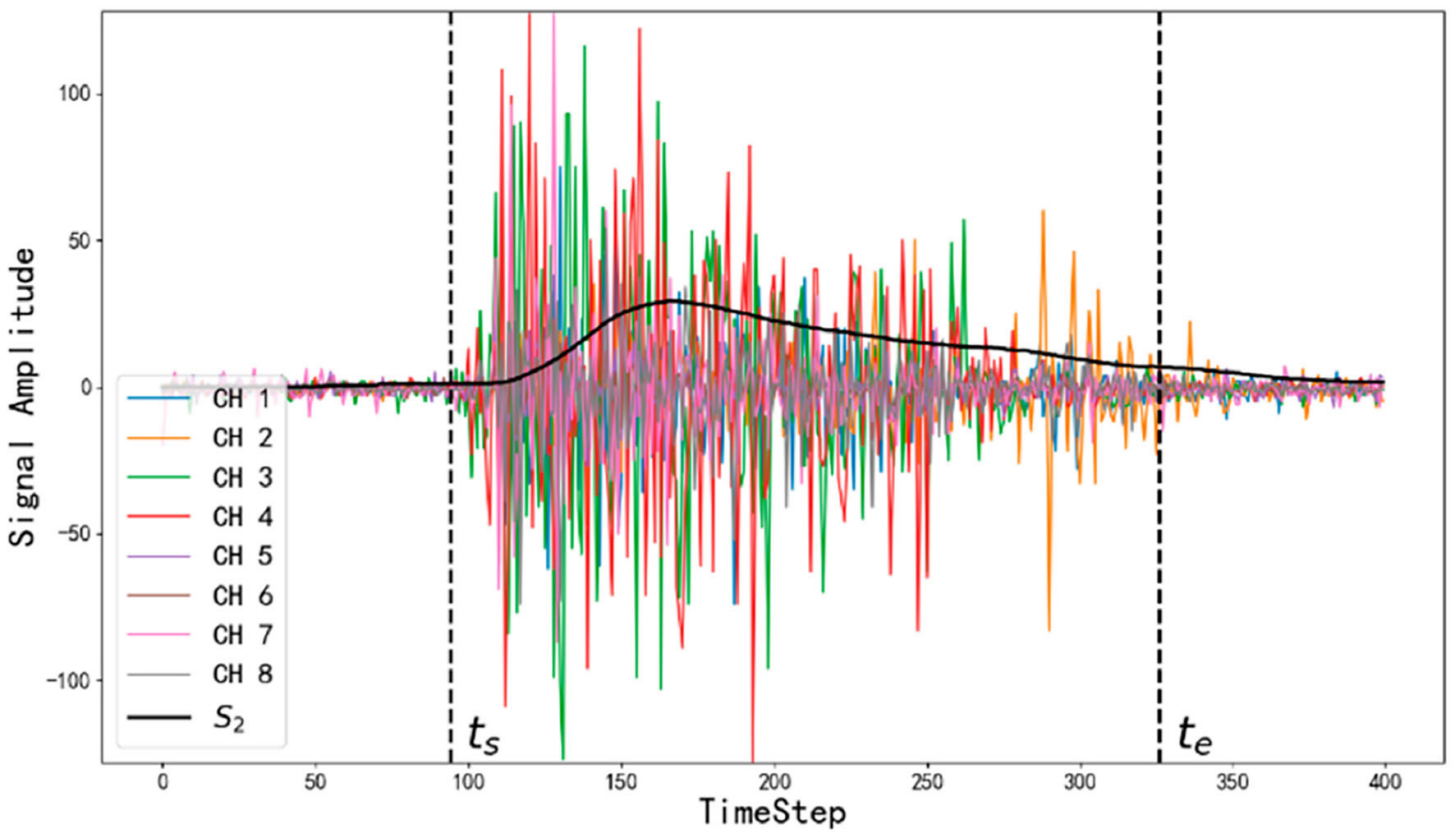
The curves of surface electromyography (sEMG) and the motion detection result (Zhang et al., 2020).

In order to speed up training and improve the accuracy of prediction, it is usually necessary to detect the region of the sEMG corresponding to gesture, in other word, muscle activity, and removing signals where the muscles are rest. A threshold should be determined, and then all the instants greater than or equal to the threshold are extracted from the smoothed signal. The first of these instants is the beginning of the muscle activity region, and the last instant is the end of the activity region (Zhang et al., 2011, 2020; Benalcázar et al., 2017; Qi et al., 2020). As depicted in Figure 3, CH1-8 are eight channel sEMG signals, and *S*_2_ is the standard deviation of eight channel sEMG signals calculated by moving average method.

In the field of prosthetic hand sEMG control, the optimal sliding window length can guarantee the minimum classification error with suitable controller delay (Hudgins et al., 1993). Too long sequence may cause system long processing delay, while too short window may not contain enough useful information. For a reliable prosthetic hand control system, the maximum allowable delay between signal generation and drive command generation should not exceed 300 ms (Hudgins et al., 1993). Study in Englehart and Hudgins (2003) show that 150–250 ms windows for sEMG are the best for mechanical sensors. Nielsen et al. (2011) proved that the performance of the system will decline when the sliding window length is <100 ms. Windows may be either disjoint or overlapped. Real-time continuous classification not only needs high classification accuracy but also requires rapid response (Huang et al., 2009). The overlapping analysis window method can accelerate the decision (Englehart and Hudgins, 2003). The key to the scheme is to set the window increments carefully. In terms of hardware processing power, overlapping analysis windows that generate fast and dense decision flows are usually preferred.

### 2.3. Feature Extraction

In order to achieve better accuracy and robustness, DL techniques needs not only a good algorithm but also a good input. There are two input methods: (1) traditional manual feature extraction method is used to increase the data density of sparse multi-channel sEMG; (2) the raw sEMG signal is directly input into the network to realize end-to-end learning.

#### 2.3.1. Traditional Feature Extraction Method

For the traditional feature extraction methods, many researchers are devoted to propose new features based on professional knowledge (Khushaba et al., 2017), or to present new feature sets by analyzing existing features (Phinyomark et al., 2009, 2012a; Bi et al., 2019). The goal of feature extraction is to increase the information density implicit in sEMG signals, and improve the difference between gestures (Oskoei and Hu, 2007). The classical features contain considerable heuristic knowledge; thus, integrating these classical features with DL approaches could improve the gesture recognition performance on sparse multichannel sEMG. The existing sEMG features can be divided into four types: time domain features (TD), frequency domain features (FD), time-frequency domain features (TFD), and parameter model (Oskoei and Hu, 2008; Phinyomark et al., 2013; Jali et al., 2015; Nazmi et al., 2016).

***Time domain features:*** The TD values are calculated directly from the raw sEMG, which are functions of time. Compared with other features of sEMG, they have low computational complexity and have been widely used. There are no <27 time domain features of sEMG, but most of them are redundant. Time domain features constantly used consist of waveform length (WL), mean absolute values (MAV), integrated EMG (iEMG), histogram (HIST), root mean square (RMS), zero crossings (ZC), standard deviation (SD), slope sign change (SSC), Willison amplitude (WAMP), variance (VAR), V-order (V), simple square integral (SSI), and so on, which are often used in combination. The detailed information is shown in Table 1.

**Table 1:** Features according to the domain.

**Category**	**Feature**	**Formula**	**Explanation**
Time domain	WL	$WL=\overset{N-1}{\underset{i=1}{\sum }}|x_{i+1}-x_{i}|$	Comprehensive information about frequency, duration and amplitude of signal
	MAV	$MAV=\frac{1}{N}\overset{N}{\underset{i=1}{\sum }}|x_{i}|$	Indication of muscle contraction levels
	iEMG	$iEMG=\overset{N}{\underset{i=1}{\sum }}|x_{i}|$	An onset detection index
	RMS	$RMS=\sqrt{\frac{1}{N}\overset{N}{\underset{i=1}{\sum }}x_{i}^{2}}$	A feature modeled as amplitude modulated Gaussian random process
	ZC	$\begin{array}{c} ZC=\sum_{i=1}^{N-1}f\left(\Delta_{i}\right),\Delta_{i}=\left|x_{i+1}-x_{i}\right|\\ f\left(\Delta_{i}\right)=\{\begin{array}{l} 1,\;x_{i}x_{i+1}<0\text{ and }\Delta_{i}>th\\ 0,\;ot \end{array} \end{array}$	A feature that provides an approximate estimation of frequency domain properties
	V	$V={\left(\frac{1}{N}\overset{N}{\underset{i=1}{\sum }}x_{i}^{v}\right)}^{\frac{1}{v}}$	A non-linear detector for implicit estimation of muscle contraction force
	SSC	$\begin{array}{c} SSC=\sum_{i=2}^{N-1}f\left(\Delta_{i}\right),\\ f\left(\Delta_{i}\right)=\{\begin{array}{l} 1,\;\Delta_{i}\geq th\\ 0,\;ot \end{array},\\ \Delta_{i}=\left(x_{i}-x_{i+1}\right)\times \left(x_{i}-x_{i-1}\right) \end{array}$	A method that represent the frequency information of signal
	VAR	$VAR=\frac{1}{N-1}\overset{N}{\underset{i=1}{\sum }}x_{i}^{2}$	A power index of the sEMG signal
	WAMP	$\begin{array}{c} WAMP=\sum_{i=1}^{N-1}f\left(\Delta_{i}\right),\\ f\left(\Delta_{i}\right)=\{\begin{array}{l} 1,\;\Delta_{i}>th\\ 0,\;ot \end{array},\\ \Delta_{i}=\left|x_{i+1}-x_{i}\right| \end{array}$	Indicatorof the level of muscle contraction
	SSI	$SSI=\overset{N}{\underset{i=1}{\sum }}x_{i}^{2}$	An energy index of the sEMG signal
Frequency domain	TP	$TP=\overset{M}{\underset{j=1}{\sum }}p_{j}$	An aggregate of the sEMG power spectrum
	MP	$MP=\frac{1}{M}\overset{M}{\underset{j=1}{\sum }}p_{i}$	An average power of the EMG power spectrum
	MNF	$MNF=\frac{\overset{M}{\underset{j=1}{\sum }}f_{j}p_{j}}{\overset{M}{\underset{j=1}{\sum }}p_{j}}$	Average frequency
	MDF	$\overset{MDF}{\underset{j=1}{\sum }}p_{j}=\overset{N}{\underset{j=MDF}{\sum }}p_{j}=\frac{1}{2}\overset{M}{\underset{j=1}{\sum }}p_{j}$	A frequency that divides the sEMG power spectrum into two equal amplitude regions

*Notations: N is the length of sampling points, x_i_ is the sEMG signal of the ith sampling point, M is total number of the frequency bin, p_j_ is the sEMG power spectrum of jth frequency bin, f_j_ is frequency of the spectrum of jth frequency bin, th is threshold, and ot is otherwise*.

***Frequency domain features:*** The FD features are calculated by Fourier transform of the autocorrelation function of sEMG signal and estimated by periodogram or parameter method. The common frequency domain characteristic of sEMG signal is frequency ratio (FR), total power (TP), mean power (MP), median frequency (MDF), mean frequency (MNF), power spectrum (PS), and so on. The detailed information is shown in Table 1.

***Time-frequency domain features:*** The TFD features are expressed as locating the energy of sEMG signal both in time and frequency, which is usually an important approach in feature extraction. The typical representative of time-frequency analysis is wavelet transform (Joshi et al., 2015). Different wavelet coefficients constitute different frequency bands and statistical index are extracted as TFD features. When db4 is used as the wavelet basic function, the raw sEMG signal is decomposed with the level of 3 and the wavelet transform equation is defined as:

$$WT=\frac{1}{\sqrt{a}}\int_{-\infty }^{+\infty }f(t)\psi^{*}\left(\frac{t-\tau }{a}\right)\text{d}t$$

where *a* is the scale parameter, ψ(*t*) is the mother wavelet, and τ is the translation parameter. *n*_*i*_ is the length of the *i*th level decomposition coefficient *c*_*i*_ (*c*_*i*_ = one of [*cD*1, *cD*2, *cD*3, *cA*3]); μ is the mean of *c*_*i*_ (Liang et al., 2019). In this case, the typical TFD features include mean of the absolute coefficient *c*_*i*_ (MOAC_*i*_), average power of the coefficient *c*_*i*_ (APOC_*i*_), standard deviation of the coefficient *c*_*i*_ (STDOC_*i*_), ratio between MOAC_*i*+1_ and MOAC_*i*_.

***Parameter model:*** The basic principle of the parameter model is to regard it as a time series based on sequence information in the raw sEMG. Because sEMG signal is steady on short notice, the coefficients and intercept of the fourth-order autoregressive model are often selected as the characteristic values (Phinyomark et al., 2012b).

#### 2.3.2. sEMG Image

**Figure 4:**
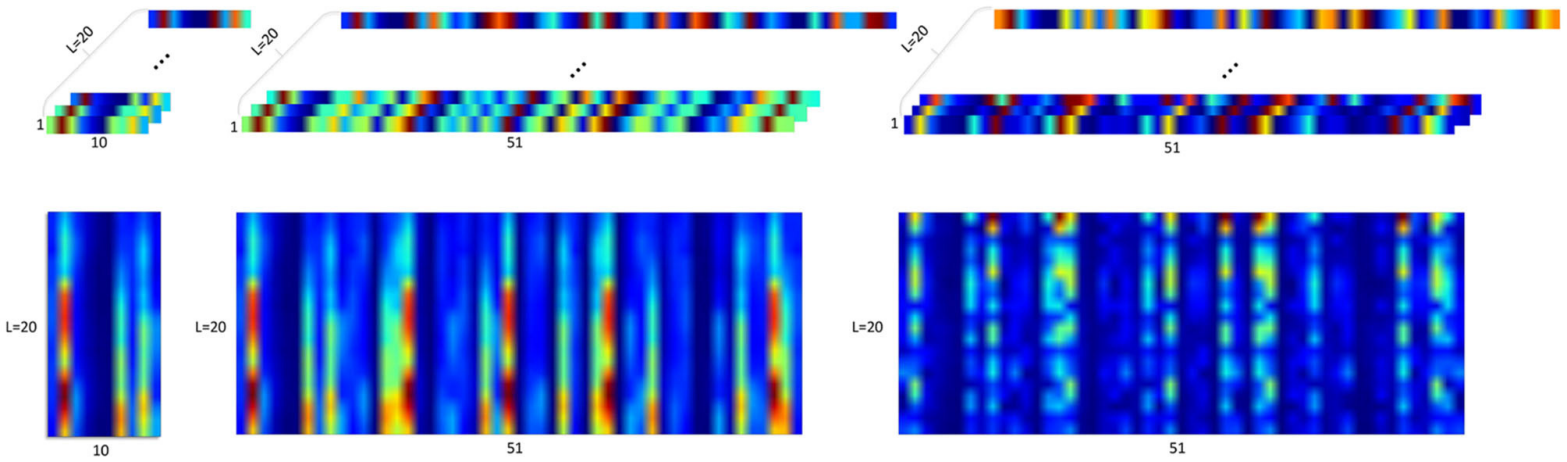
Three surface electromyography (sEMG) image representation methods based on raw signal (Hu et al., 2018).

Inspired by the spatiotemporal characteristics implied in the HD-sEMG and the end-to-end learning characteristics of DL, the method of hand gesture recognition using the sEMG image formed from the raw sEMG was proposed. The two-dimensional array distribution of electrodes in HD-sEMG allows us to analyze sEMG information in spatial domain, which makes it possible to analyze sEMG signals using image processing techniques. The pixels of sEMG image can be defined by the distribution of electrode array, that is, the row and column of electrode. This creates an image with size *L* × *W* × *H*, where *L* is the number of frames and the number of color channels of the sEMG image, *W* is rows of the array electrode and the width of sEMG image, and *H* is columns of the array electrode and the height of sEMG image (Geng et al., 2016). There are four kinds of image representation methods of raw sEMG signal, which are composed of raw-image, signal-image, activity-image, and feature-signal-image (Hu et al., 2018). The input of these method is a time-window sEMG signal whose size is *L* × *C*, where *L* is the length of the time window or the number of color channels of sEMG image, and *C*is the number of signal channels or the height of sEMG image. The **raw-image** method is gained by transforming the input signals into images with size *L* × *W* × *C* or *W* × *L*×*C*, where *W* is the width of sEMG image and *width*×*height* = *signalchannels* (Atzori et al., 2016; Zia ur Rehman et al., 2018b). The **signal-image** is obtained by realigning the information of each channel with size *L*×*W*×*C* or *W* × *L* × *C*. The **activity-image** is formed by fast Fourier transformation of signal-image with size *L* × *W* × *C* or *W*×*L*×*C*. The **feature-signal-image** is based on the signal image to process the traditional feature extraction method for each channel signal, whose size is *F* × *L* × *C*, where *F* is feature set selected and *featuredimension* = *signalchannels* × *featurevectordimension*. Figure 4 is a representation of the first three sEMG images. An instantaneous sEMG image is a single sample of a motor unit action potential distribution under an electrode grid at a specific time, which could reduce the time required to process the signal. The number of instantaneous sEMG images captured per second is the sampling frequency in time.

### 2.4. Classification

#### 2.4.1. Convolutional Neural Network

Convolutional neural network (CNN) is the most extensive applications for DL architecture based on sEMG gesture recognition. Park and Lee (2016) proposed a user-adaptive CNN model, which is the first DL-based architecture applied to sEMG signals, to classify data from the Ninapro database resulting in a better classification accuracy and robustness of variability compared to support vector machine. Atzori et al. (2016) introduced a modified version of the well-known CNN architecture, called LeNet, to classify an average of 50 hand movements in NinaPro database. The results show that CNN could produce more accurate classification accuracy compared to traditional techniques (Linear Discriminant Analysis, Support Vector Machine, and k-Nearest Neighbor). Consider the correlation between specific muscles and gestures, Wei et al. (2019) adopted a “decomposition-and-fusion” approach for gesture recognition based on sEMG, which was called two-stage multi-stream CNN strategy and proved that the performance of multi-stream CNN is better than random forest and simple CNN. Chen J. et al. (2020) took advantage of 3D CNN in processing the data that was implanted in the finger movement to process HD-sEMG. Due to too many parameters of CNN model, Chen L. et al. (2020) designed a compact network structure, which cannot only effectively reduce the complexity of the model but also improve the accuracy of prediction. Based on several fundamental factors, such as pre-processing, hyperparameters, and network structure, Park and Lee (2016), Tsinganos et al. (2018), Asif et al. (2020), Chen L. et al. (2020), and Triwiyanto et al. (2020) studied the techniques of optimizing the performance of CNN model. There are other CNN-based researches for sEMG center at improving the scalability of the model through domain adaptation (Du et al., 2017) or self-recalibrating classification (Kyranou et al., 2018). CNN also performs well in synchronous sEMG recognition (Ameri et al., 2018). There are also many papers that apply CNN architecture to embedded systems (Côté Allard et al., 2016; Tam et al., 2020).

#### 2.4.2. Recurrent Neural Network

One of the most important advantages of CNN is that it can automatically learn spatial features from input data or extract unlabeled features. However, the sEMG is the form of time series signal essentially, which is more suitable for recurrent neural network (RNN) that has dominant position in processing temporal or otherwise sequential information (Hu et al., 2018). Simao et al. (2019) compared the performance of RNN and feed-forward neural network (FFNN) on the recognition of hand gestures. It is proved that the dynamic models (RNN) with fewer parameters can achieve similar accuracy to the static model (FFNN), and the training and inference time is shorter. He et al. (2018) propose a model combining long short-term memory (LSTM) network and multiplayer perceptron (MLP) for feature learning and classification of sEMG signals. The former captures the temporal dependence of sEMG signals, while the latter focuses on static characteristics. The accuracy of motion classification is improved by constructing a feature space containing dynamic and static information of sEMG signals. Nasri et al. (2019) studied the performance of gated recurrent unit (GRU) in hand gesture recognition approaches for first time. Zhang et al. (2020) proposed a novel RNN model with short delay for gesture recognition, which could generate an instantaneous prediction after the start of gesture motion at each sampling time step. RNN is also widely used for continuous limb motion estimation (Bengoetxea et al., 2014; Xia et al., 2018; Wang et al., 2020).

#### 2.4.3. Combination of CNN and RNN

The hybrid CNN–RNN architecture can obtain excellent performance in spatial and temporal features, while CNN or RNN can extract only one kind of feature of sEMG. Hu et al. (2018) directly connected LSTM and CNN into a unified structure, and utilized the raw sEMG as input signal for dynamic recognition of gestures. Wu et al. (2018) proposed LCNN model, which first leveraged LSTM to preserve the temporal information of sEMG to reduce the loss of temporal information, and follow CNN was used to extract spatial feature information. Tong et al. (2019) introduced a dual-flow network structure. The upper layer was a multi-layer CNN structure, and the lower layer was composed of multiple LSTMs. By learning the fusion information of the two layers, the model can extract the spatiotemporal features efficiently.

#### 2.4.4. Temporal Convolutional Network

In order to obtain higher classification accuracy and lower performance degradation in multi-day sessions, DL models often have many layers and neurons, and are based on complex architecture (Bahador et al., 2020). Recently, temporal convolutional network (TCN) has been gradually applied to the research work for sEMG-based gesture recognition, which takes advantage of a one-dimensional convolution layer running along the time dimension to learn the time dependence of a given input signal. Betthauser et al. (2019, 2020) utilized the history of sEMG signals to discover the temporal features, which improves the accuracy and stability of motion intention prediction, especially during the transitions between classes. Tsinganos et al. (2019) achieved high accuracy that surpasses the result obtained with CNN on the 53 classes tasks. Zanghieri et al. (2020a,b) apply TCN to embedded devices and prove that the performance of TCN is better than the classical ML method in terms of the limited power budget and computing resources.

#### 2.4.5. Unsupervised Learning

The methods mentioned above, including CNN, RNN, and TCN, are all supervised learning approaches. In the pattern recognition techniques based on sEMG, these approaches are dominant; however, unsupervised learning methods have been gradually applied to this field in recent years. The application of supervised learning involves a labeled training dataset, while unsupervised learning approach has minimal or no requirement for data labeling, potentially reducing human errors in annotation. At present, unsupervised learning approaches in DL techniques are mainly divided into two categories: one is autoencoders (AE), whose goal is to recover the original data as lossless as possible from the abstract data; the other is probabilistic restricted Boltzmann machine (RBM) and its improved algorithm deep belief network (DBN), whose goal is to maximize the probability of the original data appearing when the RBM reaches a stable state. AE are data compression algorithm, which use efficient data encodings to map noisy input data into clean output (Le, 2015). Vujaklija et al. (2018) used AE and advanced industrial control algorithm to realize the sEMG control task of upper limb with 2 DOFs, which effectively proved that the performance of AE slightly outperformed the state-of-the-art factorization algorithms, i.e., non-negative matrix factorization. Zia ur Rehman et al. (2018a) evaluated the performance of AE and LDA using multi-session sEMG data from the healthy and amputees, and proved that AE could significantly improve the performance of the pattern recognition techniques based on sEMG. Zia ur Rehman et al. (2018b) compared CNN and AE on a multi-session dataset. The results showed that AE outperformed CNN in intra-session analysis, but CNN had stronger robustness and higher computational efficiency. DBN is a generation model composed of explicit neurons and implicit neurons, in which the former are used to receive signal input and the latter are used to extract features. By training the weights of each neuron, it can automatically learn features to realize signal recognition and classification. Shim and Lee (2015) proposed using DBN to recognize multi-channel EMG signals, and found that the classification performance of DBN was better than LDA, SVM, and BP. Zhang J. et al. (2019) developed DBN to deal with the non-linear and time-varying properties of sEMG signal, and proved the potential of DBN through the measured data. Although one of the advantages of unsupervised learning is that it eliminates the need for manual tagging, self-training strategy is likely to increase classification errors, especially with data distribution mutation (Huang et al., 2017; Côté-Allard et al., 2020).

#### 2.4.6. Other DL Methods

Apart from the above methods, there are many other DL methods applied to sEMG pattern recognition. Because of the simplicity of the structure, fully connected neural networks (Neacsu et al., 2019), BP neural network, and artificial neural network (ANN) (Mane et al., 2015; Liu et al., 2017; Yang and Zhang, 2019; Zhang Z. et al., 2019) are also commonly used for offline and real-time identification of sEMG. Time delay neural network (TDNN) is an established neural network architecture for time series processing. TDNN uses tapped-delay lines to process the temporal range of key features in the input signal to provide a short-term memory that can be fed into the traditional feed-forward network architecture (Waibel et al., 1989). TDNN has two advantages. First, it does not rely on repeated isometric contractions and can be used to identify instantaneous muscle contractions based on natural motion. Second, it can recognize the motion of 2 DOFs at the same time (Smith et al., 2009). TDNN processing of combined with sEMG and kinematics data could show excellent performance in the prediction of simultaneous motion of shoulder joint and elbow joint (Kwon and Kim, 2011; Blana et al., 2016; Day et al., 2020). Since the quantity of training datasets in DL affects the performance of the model, the limited datasets collected from multiple topics should be first extend by certain data-augmentation approaches that can also enhance the robustness of the model (Tsinganos et al., 2018; Yang et al., 2018; Côté-Allard et al., 2020). Moreover, due to the nature of the training in a neural network, the process of transfer learning is very straightforward (Yosinski et al., 2014; Côté-Allard et al., 2019). Table 2 presents a summary about the structure of some models in this review.

**Table 2:** Standard structure used by the selected models.

**Paper**	**Dataset**	**Subjects/sessions**	**Classes/channels**	**Time window**	**Feature**	**Classification**	**Accuracy (%) Intra/Inter**	**Real-time**
Park and Lee (2016)	NinaPro DB1	27/1	6/10	2,000 ms	RMS *time* × *ch*.	CNN	N.A./94	No
Atzori et al. (2016)	NinaPro DB1, DB2, DB3	78/1	52/10	150 ms	mDWT, HIST, WL, RMS	CNN	N.A./66.59, 60.27, 38.09	No
Tsinganos et al. (2018)	NinaPro DB1	27/1	53/8	200 ms	RMS *time* × *ch*.	CNN	70.5/N.A.	No
Chen L. et al. (2020)	Private	17/1	5/8	260 ms	CWT	CNN	98.81/N.A.	No
Asif et al. (2020)	Private	18/1	10/6	150 ms	Raw sEMG	CNN	92/N.A.	No
Triwiyanto et al. (2020)	Private	10/1	10/2	200 ms	Raw sEMG	CNN	93/N.A.	No
Du et al. (2017)	CapgMyo DBb	8/2	8/128	1 sample	Instantaneous sEMG	CNN	98.6/63.3	Yes
Chen J. et al. (2020)	CapgMyo DBa	23/1	8/128	1 sample	Instantaneous sEMG	CNN	98.9/N.A.	No
Wei et al. (2019)	CapgMyo DBa	23/1	8/128	1 sample	Instantaneous sEMG	CNN	99.8/N.A.	No
He et al. (2018)	NinaPro DB1	27/1	52/10	400 ms	Raw sEMG	LSTM	75.45/N.A.	No
Simao et al. (2019)	NinaPro DB5		8/16	500 ms	Standard deviation	RNN	92.07	Yes
Nasri et al. (2019)	Private	35/1	6/8	37.6 ms (4 ms)	Raw sEMG	GRU	80/N.A.	No
Hu et al. (2018)	NinaPro DB1	27/1	53/8	200 ms	RMS	CNN + RNN	87.0/N.A	No
Wu et al. (2018)	Private	4/1	5/8	260 ms (25 ms)	Raw sEMG	LSTM + CNN	98.14/N.A.	No
Tong et al. (2019)	Private	8/1	5/18		SampEn, ZC, MAV	Dual-Flow Network	78.31/N.A.	No
Betthauser et al. (2019)	Private	9/1	27/8	1675 ms	20 ms-MAV	TCN	69.5/N.A.	No
Tsinganos et al. (2019)	NinaPro DB1	27/1	52/10	300 ms, 2,500 ms	RMS	TCN	89.76/N.A.	No
Zanghieri et al. (2020a)	Private	3/20	9/8	150 ms	Raw sEMG	TCN	97.1/93.7	Yes
Betthauser et al. (2020)	Private	15/1	3/8	200 ms (25 ms)	MAV, WL, VA, SSC, ZC	ED-TCN	72.1/N.A.	No
Zanghieri et al. (2020b)	NinaPro DB6	10/10	7/14	150 ms	RMS	TCN	71.3/65.0	No

### 2.5. Post-processing

In recent years, a massive amount of research on the robustness and usability of sEMG control system. The post-processing techniques play a very important role in this condition (Yu et al., 2020). When the classifier is used to decode sEMG signals from different finger movements, the post-processing techniques are usually used to prevent the prosthesis controller from being overloaded due to a host of classification instructions, and to gain better performance of the classifier by eliminating the wrong classification caused by unexpected actions (Englehart and Hudgins, 2003; Zhang et al., 2017; Samuel et al., 2019). The commonly used post-processing techniques could be roughly divided into three categories. The first strategy is to use multiwindow joint decision-making, which is based on the consistent decision of continuous multi-windows to correct some window errors. This method includes majority vote (Tam et al., 2020), elimination of consecutive repetitions (Benalcázar et al., 2018; Simao et al., 2019), threshold (Chung and Benalcázar, 2019; Yang and Zhang, 2019), and Bayesian Fusion (Khushaba et al., 2012). The second strategy is to use the confidence score to determine the decision, i.e., to improve the classification accuracy by rejecting unreliable decisions (Scheme et al., 2013). The third is to tolerate some error classification directly and adopt special control strategy to reduce the influence of error decision (Simon et al., 2011).

### 2.6. Performance Evaluation

#### 2.6.1. The Metrics Used to Evaluate Models

The accuracy of prediction generally is one of the leading criterion that is employed to evaluate the functionality of a pattern recognition model. However, when evaluating the performance of the whole pattern recognition techniques based on sEMG, more factors should be considered. In offline testing, the performance of the model is generally evaluated by accuracy, recall, precision, standard deviation, etc. (Mane et al., 2015; Benalcázar et al., 2018; Chung and Benalcázar, 2019; Yang and Zhang, 2019; Zhang Z. et al., 2019). And these measures usually are classified as error measures, such as mean square error (MSE), root mean square error (RMSE), normalized root mean square error (NRMSE), and correlation coefficient (R) (Wang et al., 2020). The mathematical equations of these measures and their significance in the model are listed in Table 3. According to these metrics, the results may be biased for two reasons: when the prediction results of the model determine what gesture was carried out and when was executed and by whom, the prediction is regarded as true affirmation, but in the actual system, only what gesture when was executed is considered. In addition, the data of each category and each generator in the test set of the model should be balanced, otherwise the accuracy of the model will tend to be the same as that of the subjects with more data. When building the real-time model, the system performance measurement can measure the accuracy of the complete system in real time. These measures are based on Fitts' law (Fitts, 1954), which is used to measure the prediction model of the target performance of the designed system (Kamavuako et al., 2014; Wurth and Hargrove, 2014; Ameri et al., 2019; Waris et al., 2019; Xiao et al., 2020). These performance indicators include six metrics, such as overshoot, throughout, path efficiency, completion rate, average speed, and stopping distance (Table 4).

**Table 3:** The commonly used offline performance metrics.

**Evaluation metric**	**Mathematical definition**	**Explanation**
Accuracy	$Accuracy=\frac{TP+TN}{TP+FP+TN+FN}$	It is the proportion of the corresponding gesture recognized by the model in all the data.
Recall	${Recall}_{k}=\frac{TP_{i}}{TP_{i}+FN_{i}}$	It is the proportion of correctly recognized data in a gesture class.
Precision	${Precision}_{k}=\frac{TP_{i}}{TP_{i}+FP_{i}}$	It is the proportion of a class of gestures correctly recognized among the gestures recognized by the model.
*F*1_*scor*_*e*__*k*__	${F1}_{score_{k}}=2\times \frac{{Precision}_{i}\times {Recall}_{i}}{{Precision}_{i}+{Recall}_{i}}$	
Standard deviation of the accuracy per user (*SD*_*users*_)	$SD_{users}=\sqrt{\frac{\overset{n}{\underset{i=1}{\sum }}{\left(Accuracy_{user\left(i\right)}-Accuracy\right)}^{2}}{n-1}}$	It is the dispersion of each subject's recognition accuracy.
Standard deviation of the accuracy per class (*SD*_*classes*_)	$SD_{classes}=\sqrt{\frac{\overset{g}{\underset{k=1}{\sum }}{\left(Recall_{class\left(k\right)}-Accuracy\right)}^{2}}{g-1}}$	It is the amount of dispersion of the recalls of a particular model.
Mean square error (MSE)	$MSE=\overset{n}{\underset{i=1}{\sum }}\frac{{\left({ŷ}_{i}-y_{i}\right)}^{2}}{n}$	
Root mean square error (RMSE)	$RMSE=\sqrt{\overset{n}{\underset{i=1}{\sum }}\frac{{\left({ŷ}_{i}-y_{i}\right)}^{2}}{n}}$	It can be used to evaluate the numerical error of amplitude.
Normalized root mean square error (NRMSE)	$NRMSE=\frac{RMSE}{y_{max}-y_{min}}$	It is the standardization of RMSE.
Correlation coefficient (R)	$R=\frac{\overset{n}{\underset{i=1}{\sum }}\left(x_{i}-\bar{x}\right)*\left(y_{i}-ȳ\right)}{\sqrt{\overset{n}{\underset{i=1}{\sum }}{\left(x_{i}-\bar{x}\right)}^{2}*\overset{n}{\underset{i=1}{\sum }}{\left(y_{i}-ȳ\right)}^{2}}}$	It can measure the similarity between signal shapes.

*TP, true positive; FP, false positive; TN, true negative; FN, false negative. k represents the index of different classes. i represents the set of subjects. n represents the total number of subjects. k represents the set of actual classes. g represents the total number of actual classes. y represents the actual value. ŷ represents the estimated value. ȳ represents the mean of the actual value. x estimated value. $\bar{x}$ is the mean of the actual value*.

**Table 4:** The commonly used real-time performance metrics.

**Evaluation metric**	**Mathematical definition**	**Explanation**
Throughput (TP)	$TP=\frac{TD}{T_{c}}$	Quantify availability by the ratio of speed to accuracy, and defined the ratio of difficulty index of each target task to completion time.
Completion rate (CR)	$CR=\frac{NCT}{NAT}$	Describes overall success; a ratio of the completed targets to the total number of tasks attempted.
Path efficiency (PE)	$PE=\frac{SPD}{DT}*100\%$	Describes the control quality; the ratio between the shortest distance and the actual distance.
Overshoot (O)	$O=\frac{number\text{\_}of\text{\_}times\text{\_}leaving\text{\_}target}{number\text{\_}of\text{\_}targets}$	Describes the ability to stop on a target; the average number of times data appears on the target domain and then disappears in each test.
Stopping distance (SD)		Describes the ability to hold no motion the total distance traveled during the 1-s dwell time.
Average speed (AS)	$v=\frac{TL}{T_{c}}$	Illustrates the subject's gross ability to control the target; a ratio of time spent successfully completing a task to the total completion time.

*T_c_ represents the time to complete the target task. TD represents the difficulty of a task. NCT is the total number of completed tasks. NAT is the total number of attempted tasks. SPD represents the shortest possible distance. DT represents the distance traveled. TL represents the trajectory length*.

#### 2.6.2. Multi-Session Evaluation

**Figure 5:**
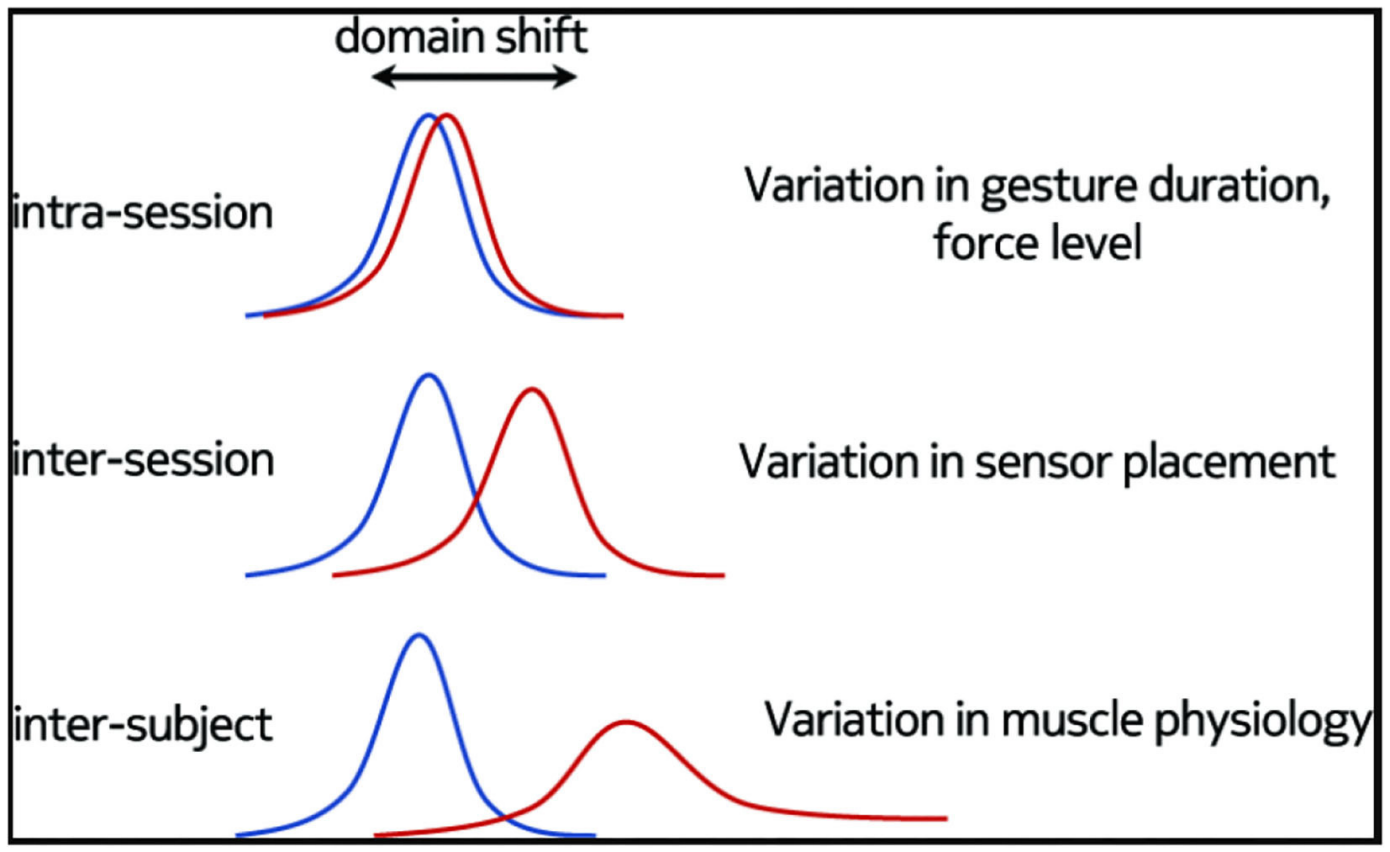
Causes of variability of data distribution (Ketyko et al., 2019).

The traditional experiment design often uses the ideal data-acquisition protocol, that is, in a conversation experiment, the subjects are asked to repeat the same gesture many times in the same session. In this case, the electrodes of sEMG sensor are kept in the same position, but the differences of time, environment and subjects in clinical and practical application are seldom considered, especially the influence of electrode insertion and removal (Yang et al., 2019). These factors would lead to different distribution characteristics of sEMG sensors data in different sessions, resulting in performance deterioration significantly with the change of sessions, and limiting the long-term use and reliability of sEMG pattern recognition techniques (He et al., 2015). The evaluation of pattern recognition techniques should not only be based on performance and computational load but also consider robustness and adaptability. From the point of view of DL, the variability in inter-session and inter-subject would lead to domain shift of the data collected by sEMG sensor, i.e., the training data and test data used by the algorithm have different distributions. The solution to this problem is to develop a new learning algorithm that can realize domain adaptation (Du et al., 2017). Domain adaptation has attracted more and more people's interest and showed a broad application prospect. In the training, when there is plenty of unlabeled data in the target domain, the existing methods generally adopt the strategy of fine tuning the pre-training model. There is also another unsupervised adaptive learning approach, which could use unlabeled target data for learning. In terms of the variability of data, the robustness and adaptability of sEMG pattern recognition techniques could be improved by multi-session evaluation, which have been usually divided into three situations (see Figure 5):

- Intra-session: Here, the model is evaluated and trained using the data from different trials in the same session of the same subject, with the electrodes not removed (Palermo et al., 2017).
- Inter-session: Here, the model is evaluated on the data from the same subject but different sessions used in the training, with the electrodes reattached (Zia ur Rehman et al., 2018b; Waris et al., 2019).
- Intra-subject: Here, the model is evaluated and trained using the data from different subjects (Ketyko et al., 2019).

## 3. Hand Gesture Recognition Challenges

In recent years, sEMG signal recognition based on DL has developed rapidly, but most of these applications are confined to improve the accuracy of offline classification and laboratory online testing, which have not been implemented in commercial systems, and even far from the real-life scenario of users. The huge gap between academia and business in this state-of-the-art technique field stems from the fact that the business community puts the actual needs of users in the dominant position of research, with clinical needs as the main purpose (Powell and Thakor, 2013; Vujaklija et al., 2017). This review has investigated the research trends in the application of DL to gesture recognition based on sEMG. We can discover that the achievements are outstanding, but there are also many challenges, which could be summed up in four categories.

### 3.1. Signal Acquisition and Processing Devices

The real-time and compactness of the prosthetic hand are the key factors affecting the user's satisfaction with the device and the use time (Biddiss and Chau, 2007), which improve demand of the requirements of sEMG data acquisition, storage, and processing. In the sEMG pattern recognition techniques of prosthetic hand, the traditional sEMG sensors can be divided into wet and dry electrodes. Wet electrode is the most commonly used electrode in clinical application. The electrode uses electrode gel for motor fixing and high-quality signal acquisition, and has good skin electrode coupling property. But there are also some shortcomings in practical application. The electrode gel may dry with time, thus changing the contact resistance between the electrode surface and skin interface. The electrode adheres to the skin and may cause skin irritation, allergy, and abrasion of the outer skin layer (Merritt et al., 2009; Pylatiuk et al., 2009). The electrode is also inconvenient to be placed in the socket of prosthetic hand (Vasluian et al., 2013). These limitations would make the currently available prostheses uncomfortable to wear and inconvenient to use. Relatively, the dry electrode is fixed on the skin by external force, which can ensure the comfort of long-term detection and high-quality signal for subsequent analysis. However, the poor contact between the dry electrode and the skin will lead to lower amplitude, higher impedance, higher randomness, and noise of the received signal (Sun and Yu, 2016; Fu et al., 2020). Recently, textile electrode has attracted more and more attention of researchers. Compared with the former two kinds of electrode, the electrode has better ventilation, flexibility, foldability, reusability, and long-term use. However, the motion artifact and noise caused by the electrode are more (Acar et al., 2019; Lee et al., 2020).

Based on the consideration of task type, control accuracy, production cost, and calculation load, the number of electrodes needed for prosthetic hand control based on pattern recognition is 8 or more (Fang et al., 2015; Rodríguez-Tapia et al., 2020). However, sEMG only contains the signal of muscle activity directly related to gesture, not enough to reflect all the information of the gesture. In order to solve these dilemmas, some research workers have come up with many ideas, such as HD-sEMG system or multi-mode sensor system including accelerometer (Jiang et al., 2018; Rizzoglio et al., 2020), near-infrared spectroscopy (Nissler et al., 2016; Scano et al., 2019), and electroencephalogram (Li et al., 2017). Accelerometer can reflect the dynamic characteristics of muscle rotation and translation. Near-infrared spectroscopy can be used to monitor muscle condition during activity. Electroencephalogram measures information related to the activity of brain nerves. However, the signal processing in existing papers relies on additional data processing hardware, such as GPU or cloud devices, which will lead to changes in the structure and energy consumption of prosthetic hand design (Nef and Riener, 2005; Kyranou et al., 2018; Sawant et al., 2020). 5G and the IoT seems to be a viable solution (Zanghieri et al., 2020a). 5G can accelerate the speed of information transfer among devices. IoT can improve the ability of collecting and sharing data.

### 3.2. Offline vs. Online Processing and Performance Evaluation

A good deal of upper limb movement pattern recognition techniques based on sEMG have been carried out in offline environment with satisfactory results. However, the system with good offline training performance does not always have excellent real-time performance (Ortiz-Catalan et al., 2015; Vujaklija et al., 2017; Gigli et al., 2020). This is because offline performance is obtained by processing offline data of sEMG. Compared with the simple offline performance indicators, the real-time performance indicators consider the amputees' reaction to the output of the algorithm and adapt to its muscle contraction to improve the application effect, which can evaluate the controllability and reliability more comprehensively (Vujaklija et al., 2017). This emphasizes the necessity of real-time assessment of pattern recognition techniques, rather than offline algorithmic performance. Most of the existing literatures focus on the pattern recognition system for upper limb amputees use of the data of intact subjects. This is because limb deficiency are unlikely to affect the amputee's motor learning ability (Tenore et al., 2009; Jiang et al., 2014b; Jarrassé et al., 2017). The study of different algorithms for intact subjects can provide a lot of valuable information to help achieve better prosthetic performance (Merad et al., 2018). Even so, it is essential to utilize the data collected from amputees (Gregori et al., 2019). Intact subjects performed real movements of the hand, while amputees could only attempt imaginary movements without visual and sensory feedback, so that when they perform the same movements at a specific arm position, the residual muscles of amputated arm might produce sEMG patterns different from those of intact arm (Geng et al., 2012; Vidovic et al., 2016). When wearing a prosthesis, the amputee's elbow, shoulder, and other parts of the body would appear different degrees of compensation movement, which could affect the sEMG signal of the hand (Geng et al., 2012; Hussaini et al., 2017; Beringer et al., 2020).

### 3.3. Individual-Specific and Variability of sEMG Signals

sEMG, as a physiological signal of muscle activation, is non-stationary in nature. Its statistical characteristics vary with time and are affected by individual differences, external factors, and many other parameters. There are many elements that could bring about the variability of sEMG signal, including physiological reasons, such as muscle condition (atrophy, hypertrophy, or fatigue), skin conditions (perspiration, humidity) (Jiang et al., 2012); user variations due to adaptation or learning (Amsüss et al., 2013); and physical reasons, such as electrode shift (Young et al., 2011), contraction intensity changes between trials, external load caused by prosthesis weight (Cipriani et al., 2011b), and arm position change (Fougner et al., 2011). This variability makes it difficult to adapt to the robustness problem in daily life, and causes the biggest dilemma in the process of long-term use for the pattern recognition techniques (Scheme et al., 2011; Park et al., 2016; Kyranou et al., 2018). From the point of view of DL, the variability in sessions and subjects would lead to different distribution of the data collected by sEMG sensor. To address these issues, new research fields have begun to analyze the real environment and focus on the validation of clinical usability (Hargrove et al., 2017; Vujaklija et al., 2017). In the experimental design, multi-user and multi-session training protocol has been adopted. In the data processing, data augmentation schemes (Boschmann and Platzner, 2012), adaptive learning (Zhai et al., 2017; Ketyko et al., 2019), transfer learning (Côté-Allard et al., 2019), and unsupervised learning (Du et al., 2017; Huang et al., 2017; Zia ur Rehman et al., 2018b) have been widely used. While these ideas are promising and encouraging, the robustness with daily variation and potential user adaptability in a new recording session over time remains unexplored.

### 3.4. Simultaneous and Proportional Information

The natural motion of the hand is continuous variation in kinematics and dynamics, which requires multiple joints or multiple DOFs to move proportionally at the same time (Muceli and Farina, 2012; Bengoetxea et al., 2015). The classification generated in sEMG-based pattern recognition is discrete and limited, which cannot produce reliable proportion information. In fact, the features of a specific pattern will migrate to another mode with the change of exercise intensity, which would lead to wrong classification, so that the accuracy of classification would be affected by the proportion information (Fougner et al., 2012). At present, proportional control is mainly realized by calculating the average power of all channels after the classification decision is made, but the training process will be more complex and longer (Muceli et al., 2014; Ameri et al., 2019). Regression methods could be used to create continuous mapping from muscle to spatial kinematics instead of classifying into discrete class labels. But the method of obtaining training data, such as mirror movements, is easy for the intact subjects, while difficult for the amputees (Nielsen et al., 2011; Hahne et al., 2014). The pattern recognition techniques do not utilize the neurophysiological knowledge of natural movements acquired in the recent decades, such as muscle synergy theory, which are promising for the development of a more natural prosthesis hand (Jiang et al., 2009). According to the muscle synergy theory, the extracted time-varying activation signals would be related to the motor commands descending to the spinal cord modulating spinal interneuron modules, which are represented by the time-invariant synergy weights (Bizzi and Cheung, 2013). Through these insights, sEMG signals can be expressed as a matrix embedded with multi-dimensional neural control information. Continuous mapping can also be realized by signal factorization, the principle of which is to extract the so-called collaborative weight and activation signal from the sEMG records (Jiang et al., 2009, 2012). There is a special factorization called non-negative matrix factorization (NMF). So far, the factorization method has been able to identify an enormous variety of motor neuron activities in voluntary upper limb movement, but it performs well in isometric contraction at constant or slowly changing forces, and its performance is limited under non-stationary conditions (Kapelner et al., 2018). Blind source separation is a factorization method different from NMF, which decomposes the recorded sEMG signal into the individual spike trains of motor neurons (Negro et al., 2016; Farina et al., 2017). The blind source separation based on convolution kernel compensation has been broadly tested (Glaser et al., 2007; Holobar and Zazula, 2007), and implemented online recently (Glaser et al., 2013). Another potential method is to decode motor neuron spike trains directly from sEMG signals and transform them into simultaneous and proportional information (Farina et al., 2014b; Levi et al., 2018). In this method, the sEMG signal is first encoded as spikes, and processed to recognize the gesture. After that, the activation signal is used to trigger the oscillator to generate motor commands for motion. Therefore, the muscle activity is directly mapped to the prosthetic hand kinematics (Pani et al., 2017; Vasquez Tieck et al., 2019).

## 4. Future Development of Gesture Recognition

The natural communication between human body and artificial hand is established from the perspective of human physiology, which is the development direction of intelligent prosthesis hand (Castellini et al., 2016). sEMG signal is a reliable method to predict human motion intention in prosthetic hand control system, because it does not require the special attention and professional skills of amputees, and the external environment interference factors will not affect its production. The prediction of upper limb motion intention based on sEMG and DL have broad application prospects. We believe, for the foreseeable future, that pattern recognition based on DL has high possibility to develop in these directions.

First of all, with the emergence of new sEMG sensors, we would use low-power analog front-end and microprocessor to develop high-precision wearable sEMG signal acquisition system, even HD-sEMG acquisition system. This would combine the flexibility of the system with good signal quality and keep a good balance between power consumption and computing power. So that the spatial resolution of sEMG signal is improved under the condition of limited resources, and fine motion recognition is realized.

Second, more open source databases would be established. These databases would include amputee data, high-quality data, musculoskeletal data, complete muscle measurement point data, upper limb kinematics data, and various application scenarios, which would promote the algorithm research of sEMG signal and attract more people to join the field of human–computer interface of prosthetic hand. Standardized sEMG acquisition protocol should be set up to further reduce the difficulty of independent experiments. Simultaneously, it is necessary to evaluate the feasibility of sEMG signal recognition and sEMG control strategy from the clinical point of view.

Third, with the further research of human joint motion and other physiological signals and the continuous development of multi-sensor fusion technique, gesture recognition based on sEMG will be paid more and more attention. Other sensors that could obtain the information of external environment or other physiological information and sEMG can complement and adjust each other to reduce the signal processing time (Fang et al., 2020). At the same time, through the appropriate mapping strategy, the calibration and refinement between different signals can be realized, which can directly reflect the process of hand movement. The multi-sensor fusion technique and the pattern recognition techniques based on DL are combined to provide the key information of continuous control for HCI interface, improve the accuracy of pattern recognition, and meet the requirements of robust recognition performance.

Fourth, the biggest problem of using DL techniques to process sEMG signal might be the absence of large-scale, labeled training data sets and resource constrained hardware platform. It is not easy to simply focus on collecting large data sets and expanding hardware resources. Instead, researchers should focus on developing techniques that make more efficient use of existing data sets. These techniques have two main directions. On the one hand, transfer learning techniques are used, so that models trained for specific scenarios can benefit from other essentially similar fields. On the other hand, different classifiers are integrated to realize the integration of different training models. When these models are combined together, more data variability would be captured in the training process, resulting in enhanced modeling ability.

Fifth, a novel functional evaluation protocol should be established. The sEMG pattern recognition model applied to prosthetic hand is used by amputee. Therefore, the evaluation of the model should consider the purpose of the use more, that is, what amputee can do now (which can be measured directly in the laboratory), what amputee can do after training (which is a reflection of behavior in life and working environment) (de Vet et al., 2011). At the level of body function, besides recording sEMG, the body kinematics should be recorded to record compensatory movement, body and cognitive fatigue, and implementation mode (Castellini et al., 2016). In the development stage of the model, the performance should be measured in the daily life of amputees before the trial stage or the system reaches its final stage based on the participation of the target population and the pilot test. The problem can be solved by using the simulation scale developed by experts. Amputees, medical staff, and researchers should be involved in evaluating the advantages and disadvantages of different pattern recognition systems and the impact of the system on different amputation levels.

## 5. Conclusions

This review paper briefly introduces the advances of DL-based sEMG pattern recognition techniques for the prosthetic hand in recent years. Through the literature survey of DL application in sEMG recognition, some of the core techniques are highlighted, some of the most common challenges to be solved are analyzed, and some of the most possible development prospects are discussed. It could be found that gesture recognition techniques based on DL has great potential in using sEMG signal to accurately interpret amputee's motion intention, which is of great significance to the development of intelligent prosthetic hand. However, their individual difference, real-time usability, and long-term stability are still being highly limited by many complex factors in these approaches. The high variability of sEMG, the lack of existing data, the limitation of hardware resources, and the lack of clinical evaluation conditions seriously affect the progress of pattern recognition techniques based on DL. In addition, the natural movements of the upper limbs are independent, continuous, and proportional activations of multiple DOFs, while the existing techniques can only use a limited number of patterns for discrete classification. These should be well-improved in the future real-time application of prosthetic hands.

## Author Contributions

WL and PS wrote this paper. PS and HY suggested the methodology. PS supervised the entire project. All authors reviewed the manuscript, read and agreed to the published version of the manuscript.

## Conflict of Interest

The authors declare that the research was conducted in the absence of any commercial or financial relationships that could be construed as a potential conflict of interest.
